# Integrating tongue coating microbiome, tongue coating metabolomics, and exhaled breath metabolomics via machine learning to identify chronic atrophic gastritis

**DOI:** 10.3389/fmicb.2026.1910978

**Published:** 2026-08-03

**Authors:** Xiaofen Hou, Huanqing Xu, Long Zhu, Shanshan Ding, Yanfeng Shao, Mengting Zhang, Xuejuan Lin

**Affiliations:** 1 College of Traditional Chinese Medicine, Fujian University of Traditional Chinese Medicine, Fuzhou, China; 2 Anhui University of Traditional Chinese Medicine, Hefei, China; 3 The Third Affiliated Hospital of Fujian University of Traditional Chinese Medicine, Fuzhou, China

**Keywords:** chronic atrophic gastritis, exhaled breath metabolites, microbial dysbiosis, multi-omics, tongue coating microbiome

## Abstract

**Background:**

Chronic atrophic gastritis (CAG) is a precancerous gastric condition with limited non-invasive diagnostic options. Alterations in the oral microbiome and its localized metabolic profiles may provide early multi-omics signatures of disease progression.

**Methods:**

We performed 16S rRNA gene sequencing of tongue coating samples, along with untargeted metabolomics of both tongue coating and exhaled breath condensate (EBC) in 139 patients, including 99 patients with CAG and 40 with chronic non-atrophic gastritis (CNAG) serving as a disease comparison group. The tongue coating microbiome, tongue coating metabolome, and systemic breath volatile profiles were comprehensively characterized. The clinical cohort was randomly partitioned into a training set and an independent validation test set in a 7:3 ratio. Unsupervised PCA algorithm was implemented for metabolic clustering, and LASSO regression was applied for cross-domain feature integration.

**Results:**

CAG patients exhibited significant tongue coating microbiome dysbiosis, characterized by the enrichment of g__Arthrobacter, Veillonella, and Streptococcus, and depletion of Prevotella and Haemophilus compared to the CNAG disease comparison group. Metabolomic profiling of the tongue coating uncovered pronounced metabolic alterations, mapped via unsupervised PCA scores (PC1: 49.5%; PC2: 5.7%), identifying 56 differential tongue coating metabolites that were predominantly upregulated and significantly enriched in alanine, aspartate, and glutamate metabolism pathways. Concurrently, EBC metabolomics mapped via PCA coordinate configurations (PC1: 18.8%; PC2: 8.2%) detected 73 differential metabolites based on unadjusted Student’s *t*-test (*p* < 0.05), with a majority (56/73) being significantly downregulated, reflecting systemic metabolic variations. To bridge these high-dimensional cross-domain interactions, an optimized 26-feature panel was filtered via LASSO regression. A generalized linear Logistic Regression classifier excelled in discriminating CAG from the CNAG disease comparison group, achieving a top-tier Area Under the Curve (AUC) of 0.8667 (95% CI: 0.748–0.954), an accuracy of 78.6%, and a sensitivity of 90.0% on the independent 7:3 test set, consistently outperforming complex non-linear ensemble tree algorithms. Calibration curve and decision curve analysis (DCA) further confirmed robust fit metrics (Brier Score = 0.1634) and substantial clinical net benefit on the held-out test cohort.

**Conclusion:**

This study establishes an advanced, regularized linear machine learning framework, demonstrating that a streamlined 26-feature panel of tongue coating and exhale profiles offers a transparent, non-invasive triage tool for chronic atrophic gastritis. These tightly synchronized local and systemic multi-source trajectories provide crucial insights into oral-gastric microbial interactions and host-microbiome co-metabolism in CAG.

## Graphical abstract

**Figure:**
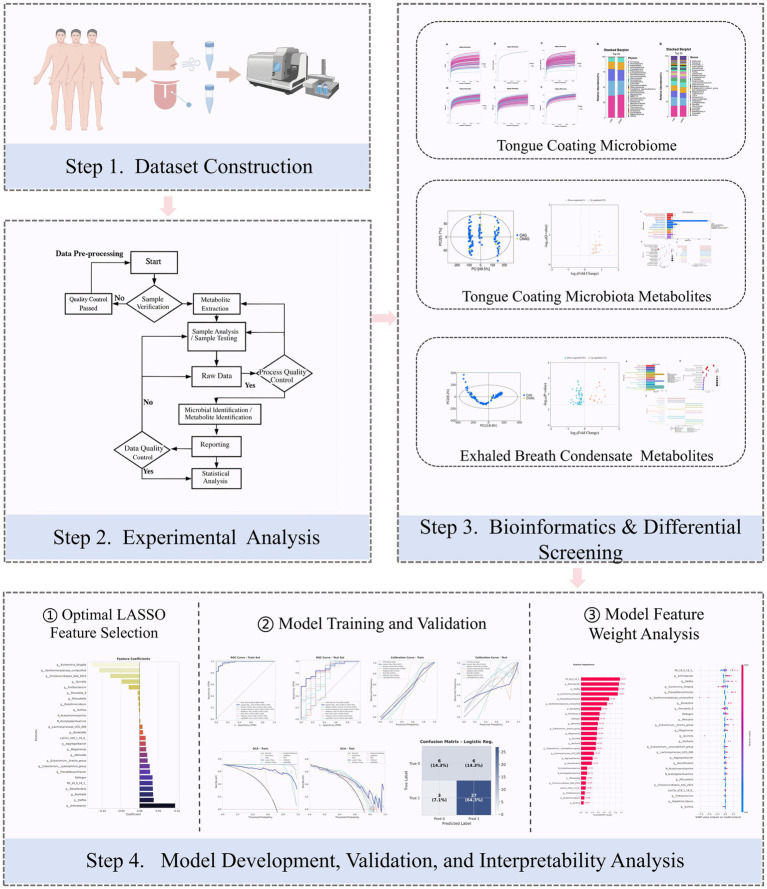

## Introduction

Chronic atrophic gastritis (CAG) is a precancerous gastric lesion that increases the risk of gastric cancer (Li et al., 2018). Early detection is critical for prevention, yet conventional endoscopic diagnosis is invasive and limited in large-scale screening (Wu et al., 2025). Recent evidence highlights the role of the oral microbiome, particularly the tongue coating, in reflecting upper gastrointestinal tract microbial ecology and potential disease-associated dysbiosis (You et al., 2024; Xia et al., 2025; Chen et al., 2025).

Moreover, systemic metabolites, such as volatile compounds in exhaled breath, may capture host-microbiome interactions and provide complementary non-invasive biomarkers (Hanna et al., 2019; Ahmadzai et al., 2013). Despite these insights, the functional interplay between oral microbial communities and metabolic alterations in CAG remains poorly understood (Hao et al., 2021; Cui et al., 2019).

In this study, we established a robust, machine learning-driven multi-omics framework by comprehensively characterizing the tongue coating microbiome, tongue coating metabolome, and systemic exhaled breath metabolome in a well-balanced clinical cohort (Xia et al., 2025; Hao et al., 2021). By integrating these high-dimensional datasets via LASSO regression and regularized linear machine learning framework (Nam et al., 2024; Asnicar et al., 2024), we aimed to: (1) map the distinctive tongue coating microbial dysbiosis and its associated localized metabolic alterations in CAG patients relative to the CNAG disease comparison group (Chen et al., 2025; Hao et al., 2021); (2) capture the systemic breath metabolic footprints that reflect underlying pathophysiological variations (Hanna et al., 2019; Ahmadzai et al., 2013); (3) construct a highly discriminative and transparent diagnostic model based on a regularized Logistic Regression formula to discover key cross-domain biomarker signatures (Nam et al., 2024; Lundberg et al., 2020); and (4) execute a post-hoc interpretable SHAP analytical pipeline to decode the internal decision logic and mathematical-biological directional alignments supporting host-microbiome trajectories. This centralized multi-omics approach not only advances our mechanistic understanding of oral-gastric microbial interactions, but also highlights a reliable, non-invasive triage tool for the screening and precision management of precancerous gastric disease (Li et al., 2018; Wu et al., 2025).

## Materials and methods

### Study cohort

To establish a methodologically rigorous and biologically homogeneous cohort for multi-omics validation, participants were screened through a structured clinical selection and age-stratified matching pipeline. Initially, a total of 318 continuous outpatients undergoing clinical screening at our endoscopy center were considered. To guarantee cross-platform data integrity, a data completeness filter was first applied, retaining only those patients who successfully completed all three independent technical evaluations [16S rRNA gene sequencing of the tongue coating, untargeted tongue coating metabolomics, and exhaled breath condensate (EBC) metabolomics], which narrowed the cohort to 139 eligible subjects. Subsequently, an age-stratified matching protocol was strictly enforced to eliminate potential age-related confounding variations in the oral microbiota and volatile organic chemical profiles, ensuring that the critical clinical baseline demographics remained highly comparable between groups. This rigorous epidemiological control naturally yielded the finalized real-world study cohort of 139 individuals, prioritising baseline homogeneity and cross-domain data synergy over simple raw sample size maximization.

From March to November 2024, a total of 139 patients with chronic gastritis were recruited at the Third Affiliated Hospital of Fujian University of Traditional Chinese Medicine, including 99 patients with chronic atrophic gastritis (CAG) and 40 with chronic non-atrophic gastritis (CNAG) serving as a disease comparison group. The clinical and pathological diagnosis of CAG strictly followed the guidelines of the 2017 China Consensus on Chronic Gastritis (National Gastroenterology Coping Group, Chinese Society of Gastroenterology, 2018), integrating clinical symptoms, gastroscopy, and histopathological evaluation. Histopathological biopsy specimens were graded for gastric atrophy, intestinal metaplasia (IM), and dysplasia according to the updated Visual Analogue Scale of the New Sydney System (Dixon et al., 1996). To ensure precise clinical stratification, the severity and topographical distribution of atrophy and IM were further staged using the Operative Link for Gastritis Assessment (OLGA) and Operative Link for Gastritis Assessment based on Intestinal Metaplasia (OLGIM) systems. Additionally, the *Helicobacter pylori* (Hp) infection status of all participants was determined via the ^13^C-urea breath test or rapid urease test at enrollment for baseline characterization; however, this covariate was not included in the subsequent multi-omics integration analysis due to the retrospective study design. The study protocol was approved by the Ethics Committee of the Third Affiliated Hospital of Fujian University of Traditional Chinese Medicine (Approval No.: 2024KS-38-1), and all procedures were conducted in strict accordance with the Declaration of Helsinki. Written informed consent was obtained from all participants prior to enrollment.

Inclusion criteria: age 18–70 years; no antibiotics, proton pump inhibitors, probiotics, or metabolic modulators within 4 weeks; written informed consent.

Exclusion criteria: Patients were excluded if they met any of the following criteria:

(1) age younger than 18 or older than 70 years;
(2) concurrent other digestive system organic diseases, including chronic enteritis, gastric ulcer, or a history of subtotal gastrectomy;
(3) severe systemic or organic dysfunctions of major organs, such as significant cardiovascular, hepatic, or renal diseases (e.g., severe diabetes, liver cirrhosis, or kidney failure);
(4) pregnancy or lactation;
(5) severe cognitive impairment or psychiatric disorders;
(6) use of the following medications within 1 month prior to sample collection: (a) oral or systemic antibiotics; (b) microecological preparations (e.g., probiotics, prebiotics, or synbiotics); (c) gastric acid suppressants [e.g., proton pump inhibitors (PPIs) or H₂-receptor antagonists]; (d) cytokines, immunosuppressants, or cytotoxic agents; (e) corticosteroids or hormone therapies; and (f) any other medications known to influence the gastrointestinal or oral microbiota; and.
(7) active oral cavity diseases (e.g., gingivitis, periodontitis, or active dental caries) or recent invasive dental treatments.

### Sample collection

Tongue coating microbiome and metabolome: Strict donor pre-conditioning was enforced to eliminate confounding oral microenvironmental variants. Participants were instructed to avoid spicy, greasy, or irritating foods, as well as the consumption of coffee, tea, and alcohol for at least 24 h prior to sampling. Subjects were strictly prohibited from heavy smoking, brushing their teeth vigorously, or utilizing commercial mouthwashes, oral cleansers, or pigmented medications on the morning of collection to prevent the artificial striping or staining of the tongue bio-matrix.

Following a mandatory 2-h fasting window (no food, water, smoking, or chewing gum), biospecimen collection was standardly executed. Participants rinsed their mouths thoroughly with 15 mL of sterile saline to ensure basal oral cleanliness. During collection, participants were guided to naturally extend the tongue, keeping the tongue tip relaxed and resting slightly over the lower lip to avoid excessive tongue contraction or localized circulatory alteration. Tongue coating samples were gently and systematically swabbed from the mid-posterior dorsum toward the tongue tip using a sterile cotton swab to ensure full representative coverage of the anatomical site without structural omission. The gathered specimen swabs were immediately transferred into sterile microcentrifuge tubes, temporarily maintained in a low-temperature environment using portable ice packs, and programmatically transferred to a −80 °C ultra-low temperature freezer within 4 h for downstream DNA extraction and metabolomic analysis (Lamont et al., 2018; Oshima and Miwa, 2016).

Exhaled breath condensate (EBC): Prior to EBC collection, the automated condensation cooling chamber was verified and stabilized at a constant temperature of −10 °C to optimize volatile/non-volatile capturing efficiency. Following the identical 2-h fasting and stabilization window, participants were equipped with a disposable mouthpiece connected to the pre-cooled condensate collector. Subjects were instructed to maintain a relaxed, seated posture and breathe naturally utilizing a steady, peaceful tidal breathing pattern for 2–3 min. Forced deep inhalation or hyperventilation was strictly avoided, and subjects were guided to sustain a uniform exhalation phase with a subtle pause at full inspiration to maximize condensate yield, standardly capturing 1–3 mL of liquid sample. Collected EBC specimens were labeled immediately and transported at 4 °C to the metabolomics laboratory for storage at −80 °C within 4 h of collection (Ahmadzai et al., 2013; Horváth et al., 2017).

### 16S rRNA gene sequencing of tongue coating

Total DNA was extracted using a cetyltrimethylammonium bromide (CTAB)-based method. The V3–V4 region of the 16S rRNA gene was amplified with primers 338F and 806R. Amplicons were purified, quantified, and sequenced on an Illumina NovaSeq 6,000 platform (2 × 250 bp paired-end). Sequence quality filtering, chimera removal, and ASV inference were performed using QIIME2 with DADA2. Taxonomic assignment used a reference database.

### Tongue coating metabolomics

Metabolites were extracted using methanol:acetonitrile:water solvent, vortexed, ultrasonicated in an ice bath, and centrifuged at 12,000 rpm for 15 min at 4 °C (Doppler et al., 2016). The supernatant was analyzed by LC–MS on a Vanquish UHPLC system coupled with an Orbitrap Exploris 120 mass spectrometer (Dunn et al., 2011). Raw data were processed using XCMS for peak detection, retention time alignment, and normalization (Want et al., 2010). Metabolites present in <50% of samples or with >30% relative standard deviation in QC samples were excluded (Sumner et al., 2007). Differential metabolites were identified based on Student’s *t*-test with a significance threshold of *p* < 0.05, which was further controlled for multiple testing using the Benjamini-Hochberg false discovery rate (FDR) correction (adjusted *p* < 0.05).

### EBC metabolomics

EBC samples were processed similarly to tongue coating samples (Ahmadzai et al., 2013). Metabolites were extracted, centrifuged, and analyzed by LC–MS. Data processing and quality control followed the same workflow (Want et al., 2010; Sumner et al., 2007). Differential metabolites were determined based on fold change and *p*-value obtained from Student’s *t*-test (*p* < 0.05).

### Microbial diversity and differential analysis

Alpha-diversity indices (Chao1 and Shannon) were calculated to evaluate microbial community richness and evenness. Beta-diversity was assessed based on Bray-Curtis distances and visually rendered via principal coordinate analysis (PCoA). To identify differential taxa between the CAG group and the CNAG disease comparison group, the Wilcoxon rank-sum test was programmatically executed at the phylum and genus levels. Multi-test combinations were strictly controlled by adjusting the raw *p*-values using the Benjamini-Hochberg false discovery rate (FDR) correction procedure, where a false discovery rate (FDR) or Q-value < 0.05 was designated as the threshold for statistical significance.

Functional prediction: PICRUSt2 was utilized to infer microbial metabolic pathways from the inferred 16S rRNA gene data. The reconstructed functional profiles were collapsed into standard Kyoto Encyclopedia of Genes and Genomes (KEGG) pathways. Differential enrichment of functional categories between the two groups was statistically evaluated utilizing the Wilcoxon rank-sum test combined with the Benjamini-Hochberg FDR correction, with significance declared at an adjusted *p* < 0.05 to identify metabolic pathways altered in CAG.

### Machine learning model construction

To integrate cross-domain variables, the multi-omics dataset was normalized and processed using Python (v3.9). To rigorously evaluate model generalizability and prevent data leakage, the clinical cohort was first randomly partitioned into a training set (70%) and an independent validation test set (30%). Cross-domain feature selection was then executed exclusively within the training set using Least Absolute Shrinkage and Selection Operator (LASSO) regression with L1 regularization. The optimal regularized constraint parameter (lambda) was determined via a 10-fold cross-validation scheme based on the minimum mean squared error (MSE) criteria. Eight benchmark machine learning classifiers—including Logistic Regression, Support Vector Machine (SVM), Multilayer Perceptron (MLP), AdaBoost, Random Forest, Gradient Boosting, XGBoost, and LightGBM—were trained on the training split according to cross-domain biomarker identification paradigms (Li et al., 2022). Model performance was systematically evaluated and validated on the held-out independent 7:3 test set using area under the receiver operating characteristic curve (AUC), 95% confidence intervals (CI), accuracy, sensitivity, and specificity metrics. Model fitting accuracy and clinical diagnostic utility were comprehensively verified on the test cohort via calibration curves with Brier score estimations and Decision Curve Analysis (DCA).

### Statistical analysis

Normality of continuous variables was assessed using the Shapiro–Wilk test. Normally distributed variables were compared using Student’s *t*-test, and non-normal variables using Mann–Whitney U test. Categorical variables were compared using Chi-square or Fisher’s exact test. Statistical significance was defined as *p* < 0.05. Analyses were performed using SPSS v29.0 or R v4.4.3.

## Results

### Study participants and baseline characteristics

A total of 139 patients with chronic gastritis were enrolled in this study, including 99 with pathologically confirmed CAG and 40 with CNAG serving as a disease comparison group. To enhance comparability and eliminate age-related biological noise across cohorts, participants were stratified into three distinct age categories (<45, 45–55, and >55 years old). As summarized in Table 1, no statistically significant differences (*p* > 0.05) were observed between the CAG and CNAG groups regarding both mean age and gender distributions within the first two strata (<45 and 45–55 years), validating robust baseline comparability following our selection pipeline.

**Table 1:** Distribution of general characteristics of CAG and CNAG patients across age groups.

Divide into groups	Under 45 (*n* = 41)			45–55 years old (*n* = 46)			55 and above (*n* = 52)		
	CAG (*n* = 23)	CNAG (*n* = 18)	*P*-value	CAG (*n* = 34)	CNAG (*n* = 12)	*P*-value	CAG (*n* = 42)	CNAG (*n* = 10)	*P*-value
Gender (Male/Female)	15/8	12/6	0.923	15/19	4/8	0.514	22/20	10/0	0.008
Age (years)	36.43 ± 6.51	33.00 ± 7.58	0.135	51.74 ± 2.78	49.75 ± 3.94	0.128	62.07 ± 3.63	69.80 ± 4.89	0.438

Data are presented as mean ± SD or frequency. *P*-values for continuous age data were calculated using two-independent-sample *t*-test. *p*-values for categorical gender data were calculated using Chi-square test, except for the >55 years old group where Fisher’s exact test was rigorously executed due to cell frequency distribution.

Notably, within the oldest sub-stratum (>55 years old), a statistically significant gender imbalance was detected via Fisher’s exact test *p* = 0.008, driven by the absence of female subjects in the localized CNAG arm (10 males vs. 0 females). This skewed demographic cluster reflects the real-world clinical screening scenario where persistent outpatients aged over 55 rarely present with pure, non-atrophic mucosal states due to decades of physiological gastric aging. Apart from this unpreventable clinical demographic characteristic, the baseline profiles remained highly balanced, preventing potential systemic confounding interactions in subsequent high-dimensional downstream omics validations.

### Dysbiosis of the tongue coating microbiome in CAG patients

**Figure 1:**
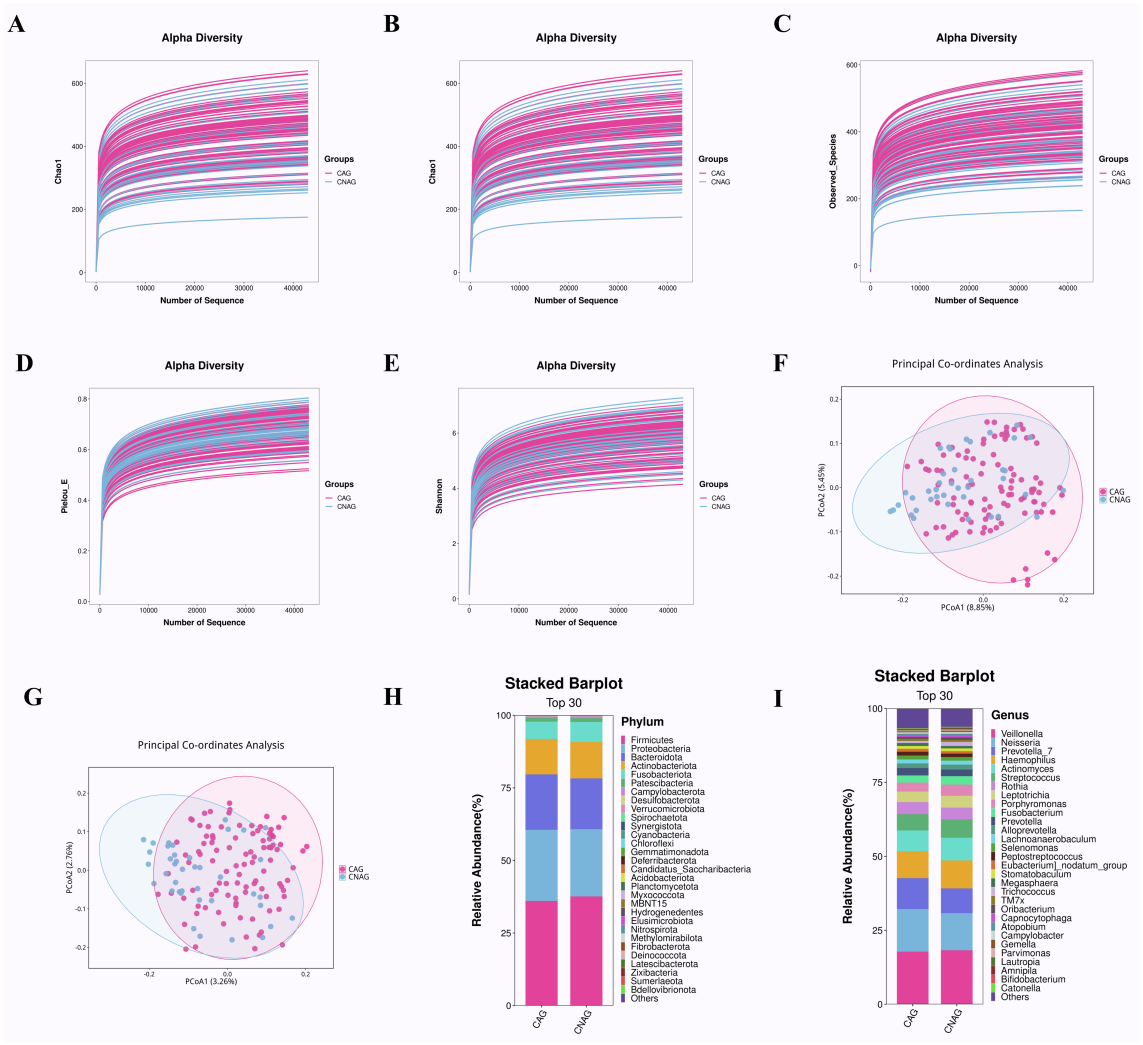
Tongue coating microbiome dysbiosis and community restructuring in CAG patients. **(A–E)** Rarefaction curves for alpha-diversity estimators, documenting sufficient sequencing depth. **(F–G)** Principal coordinate analysis (PCoA) clusters calculated from Bray-Curtis distance metrics, demonstrating substantial microecological variance between the chronic non-atrophic gastritis (CNAG) reference controls and CAG instances. **(H–I)** Stacked bar plots delineating the specific taxonomic composition and relative abundance shifts at the phylum level and top 30 genus levels, respectively.

Tongue coating microbiota analysis revealed significant changes in patients with CAG. Rarefaction curves for alpha-diversity metrics reached saturation, indicating adequate sequencing depth for downstream analyses (Figures 1A–E). Principal coordinate analysis (PCoA) based on Bray-Curtis distances showed clear separation between CAG and CNAG groups, showing significant differences in overall microbial community structure (Figures 1F,G). At the phylum level, the microbiota in both groups were predominantly composed of Firmicutes, Proteobacteria, and Bacteroidetes, collectively accounting for > 90% of total abundance. However, the CAG group showed a significant increase in Firmicutes alongside a reduction in Bacteroidetes and Actinobacteria compared with CNAG controls (Figure 1H).

At the genus level, Veillonella, Neisseria, and Prevotella were dominant across both groups. CAG was associated with significant microbial dysbiosis, characterized by enrichment of *Veillonella*, *Streptococcus*, *Lactobacillus*, and *Fusobacterium*, and concomitant depletion of *Prevotella* and *Haemophilus*, alongside enrichment of *Aggregatibacter* (Figure 1I). Differential abundance analysis identified 69 genera with significant differences (*p* < 0.05) between groups, indicating substantial restructuring of the tongue coating microbiome in CAG. Several commensal genera typically associated with oral health, including *Prevotella* and *Haemophilus*, were significantly depleted in CAG patients (Han et al., 2022; Lamont et al., 2018). In comparison, genera associated with inflammatory states or specialized metabolic niches, *Veillonella*, *Streptococcus*, and *g__Arthrobacter*, were significantly enriched (Table 2). Among these, *g__Arthrobacter* showed a particularly significant increase, underscoring its potential biological and diagnostic relevance as an indicator of localized microbial restructuring.

**Table 2:** Top 10 differentially abundant bacterial genera in tongue coating between CAG and CNAG groups.

Genus	mean_CAG	mean_CNAG	*P*-value	Regulation
*g__Arthrobacter*	0.01	0.00	*P <* 0.001	Up
*g__Desulfovibrio*	0.08	0.05	0.0001	Up
*g__Clostridiales_unclassified*	0.07	0.04	0.0001	Up
*g__Anaerotignum*	0.02	0.01	0.0001	Up
*g__Mitsuokella*	0.00	0.01	0.0001	Down
*g__Enterobacter*	0.00	0.01	0.0002	Down
*g__Alistipes*	0.02	0.01	0.0004	Up
*g__GCA-900066575*	0.00	0.00	0.0026	Up
*g__Prevotella_9*	0.04	0.06	0.0057	Down
*g__Aggregatibacter*	0.22	0.10	0.0084	Up

### Metabolomic profiling of tongue coating reveals mucosal metabolic reprogramming

**Figure 2:**
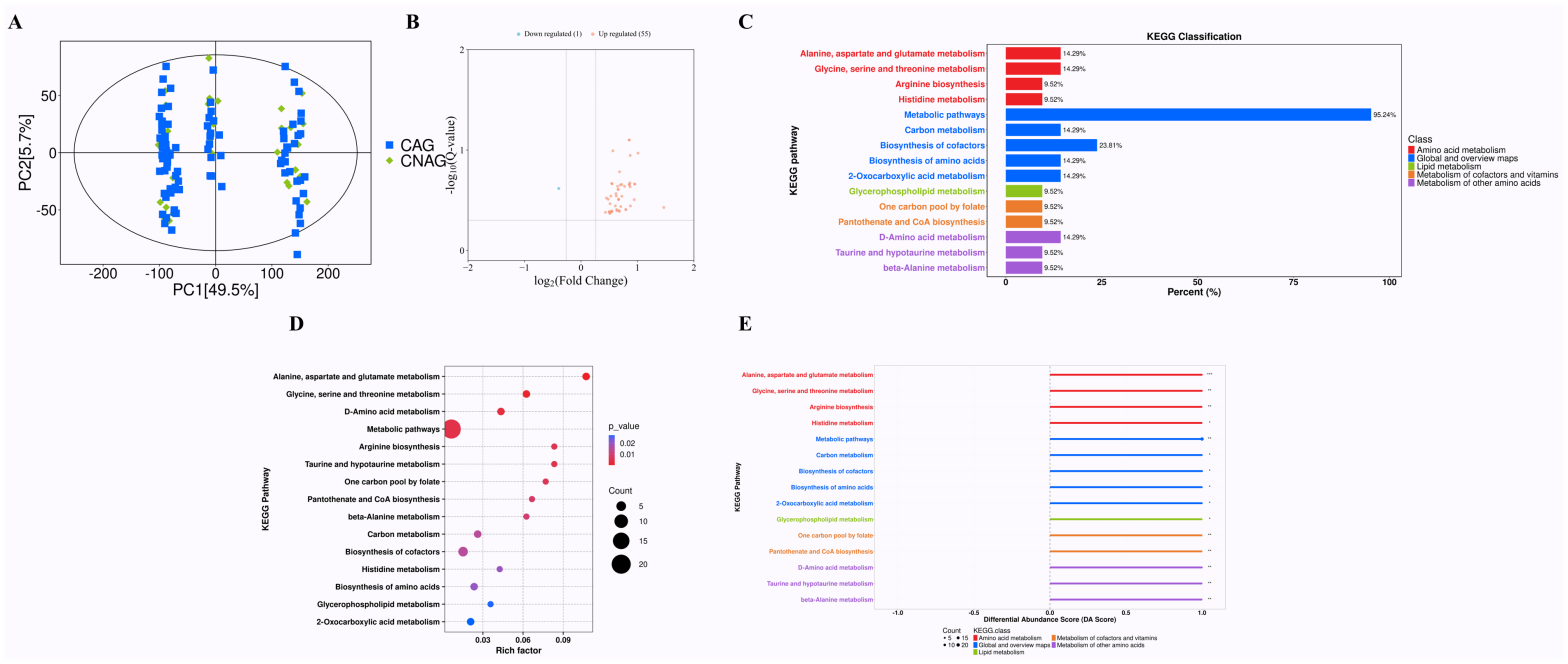
Localized mucosal metabolic reprogramming characterized via tongue coating untargeted metabolomics. **(A)** Unsupervised two-dimensional PCA score plot projected along the primary variance dimensions (PC1: 49.5%; PC2: 5.7%), delineating the natural spatial distribution based on the total cohort to map localized mucosal metabolic reprogramming. **(B)** Volcano plot profiling the distribution of differential tongue coating metabolites, with the statistical threshold defined by a False Discovery Rate (FDR) adjusted *Q* < 0.05 (horizontal dashed line) and a threshold of log2(Fold Change) > log2(1.2) (vertical dashed lines); points are color-coded to denote down-regulated (*n* = 1, light blue) and up-regulated (*n* = 55, light orange) metabolites. **(C–E)** Reconstructed KEGG classification layouts, metabolic enrichment dot plots, and Differential Abundance (DA) scores mapping enhanced amino acid turnover and pathway activation.

To further characterize the functional biochemical shifts corresponding to the dysbiotic oral-gastric microbiota, metabolomic profiling of tongue coating samples was conducted. To explore the localized biochemical variations within the oral cavity, an unsupervised Principal Component Analysis (PCA) was first applied to the tongue coating metabolic profiles, revealing distinct natural clustering trends along the top two variance axes (PC1: 49.5% and PC2: 5.7%, Figure 2A). The overlapping tendencies observed in the global PCA space precisely emphasize the high-dimensional noise and individual heterogeneity inherent in the raw profiles, demonstrating that a global supervised linear separation was limited. Therefore, using rigorous statistical selection criteria (adjusted *p* < 0.05 via Benjamini-Hochberg FDR correction),56 differentially expressed metabolites (DEMs) were identified (Figure 2B). Among these, the majority (55/56) were upregulated in the CAG group, indicating extensive metabolic reprogramming in the tongue-coating microenvironment.

These metabolites were primarily enriched in amino acids and their derivatives (e.g., glutamic acid, alanine, phenylalanine-proline), nucleotide-related compounds (e.g., inosine, 7-methylguanine), and lipid species, including phosphatidylcholine PC(14:0/14:0) (Table 3). These results suggest enhanced activity in amino acid metabolism, energy metabolism, and phospholipid biosynthesis pathways. To further interpret these alterations, KEGG pathway enrichment analysis was performed. Differential metabolites were significantly enriched in key metabolic pathways, particularly “alanine, aspartate, and glutamate metabolism” and “glycine, serine, and threonine metabolism” (Figure 2C). Enrichment factors and statistical significance confirmed robust pathway enrichment (*p* < 0.01; Figure 2D). Differential abundance (DA) score analysis further showed that all enriched pathways were upregulated in the CAG group (DA score > 0), with “alanine, aspartate, and glutamate metabolism” showing the most significant activation (*p* < 0.01; Figure 2E). These results provide strong evidence of metabolic reprogramming in the oral cavity of CAG patients, characterized by accelerated amino acid turnover and altered energy pathways, which closely mirror the mucosal inflammatory state (Hao et al., 2021).

**Table 3:** Top 10 differentially abundant metabolites in tongue coating between CAG and CNAG groups.

MS2 name	MS2 score	mean_CAG	mean_CNAG	VIP	*P*-value	regulation
*Phe-Pro*	3.94	0.068707803	0.041666172	2.126053755	0.005185285	Up
*Glutamate*	3.93	0.878444521	0.601942592	2.492493098	0.026439041	Up
*Inosine*	3.93	0.621055377	0.439145261	2.476671616	0.030408084	Up
*Betaine*	3.92	1.281022707	0.800771855	2.113563475	0.025224315	Up
*7-Methylguanine*	3.92	0.208557391	0.142961648	2.689337727	0.029323294	Up
*Val-Val*	3.9	0.042052632	0.020880627	2.349651849	0.001275195	Up
*Alanine*	3.89	0.213462539	0.146088553	2.549256074	0.004917197	Up
*Allantoin*	3.88	0.850163422	0.54210994	1.777409003	0.005738747	UP
*N-Acetylphenylalanine*	3.88	0.170097412	0.093427839	2.450908628	0.004613619	Up
*PC(14:0/14:0)*	3.77	0.216202786	0.149858588	2.015072782	0.009021933	Up

### EBC metabolomic profile reflects systemic metabolic footprint and mitochondrial stress

**Figure 3:**
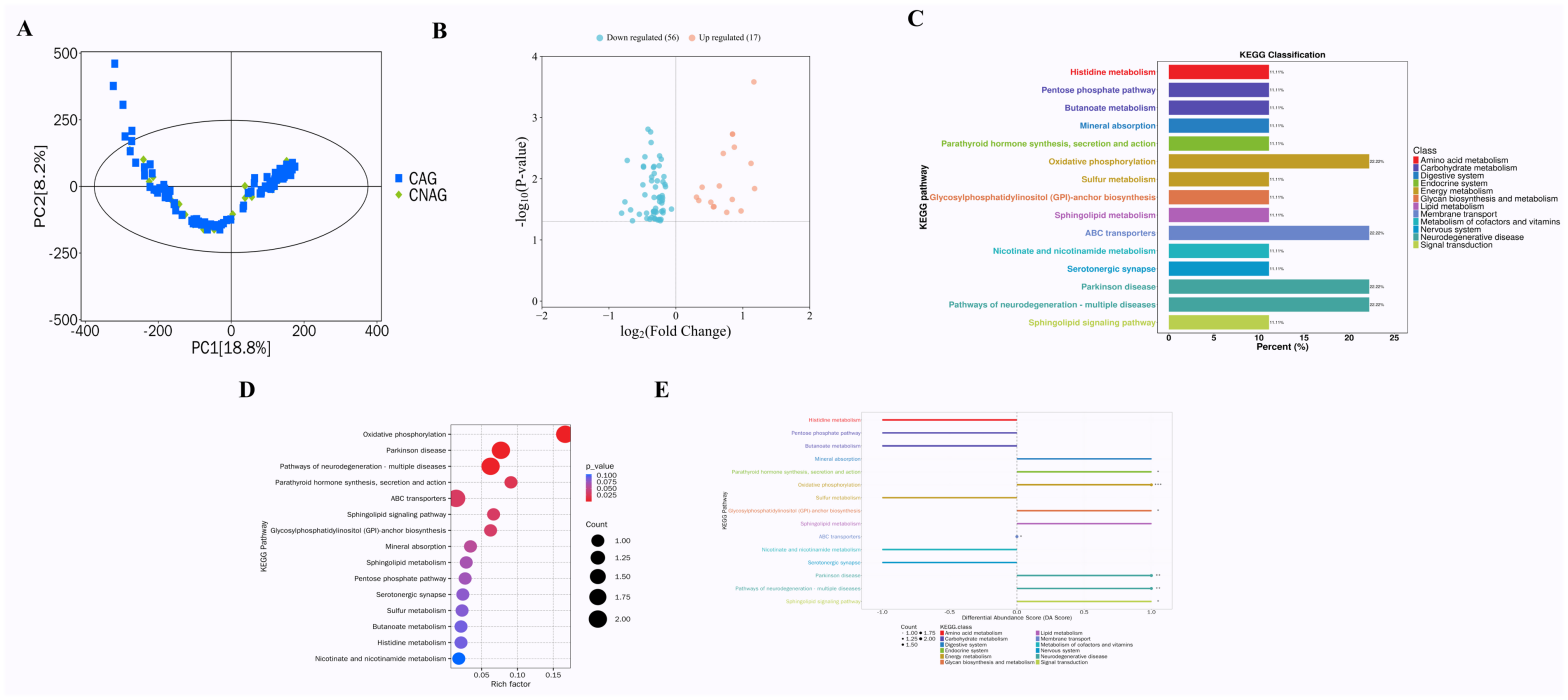
Systemic breath volatile footprints captured through exhaled breath condensate (EBC) profiling. **(A)** Unsupervised two-dimensional PCA score plot projected along the primary variance dimensions (*PC1*: 18.8%; *PC2*: 8.2%), delineating the natural spatial configuration of metabolomic features in the expired air matrix. **(B)** Volcano plot documenting targeted molecular variations, with the significance threshold defined by a raw *p*-value < 0.05 (horizontal dashed line) and directional cutoffs at log2(Fold Change) = 0 (vertical dashed line); points are color-coded to denote down-regulated (*n* = 56, light blue) and up-regulated (*n* = 17, light orange) metabolites. **(C–E)** Reconstructed KEGG classification matrices, enrichment pathway dot plots, and Differential Abundance (DA) score rankings tracking shifted mitochondrial energy stress and accelerated oxidative phosphorylation processes.

To determine whether local mucosal inflammation triggers systemic metabolic alterations, non-targeted metabolomic profiling of exhaled breath condensate (EBC) was performed. To capture the holistic metabolic variance of the exhaled breath profiles, an unsupervised Principal Component Analysis (PCA) was initially implemented, revealing distinct multi-source systemic chemical trajectories along the first two principal components (PC1: 18.8% and PC2: 8.2%, Figure 3A). In total, 73 differential metabolites were detected in EBC based on the raw Student’s *t*-test (*p* < 0.05) to evaluate baseline feature variations. Compared to the predominantly upregulated metabolic profile observed in tongue coating samples, the majority of EBC metabolites (56/73) were significantly downregulated in the CAG group (Figure 3B), suggesting a distinct pattern of systemic metabolic dysregulation or depletion during chronic disease progression.

The downregulated metabolites were highly enriched in immunomodulatory fatty acids (e.g., pentadecanoic acid, 9-octadecenoic acid) and specific volatile organic compounds (VOCs) such as decane and dodecane (Table 4). Conversely, metabolites associated with cellular energy stress and phosphorylation processes, particularly phosphates and pyrophosphates, were significantly upregulated (Table 4). KEGG pathway enrichment analysis further revealed that the differential EBC metabolites were significantly clustered in energy-associated systems. Notably, “Oxidative phosphorylation” showed the highest enrichment factor and statistical significance (*p* < 0.001; Figures 3C,D). Differential abundance (DA) score analysis confirmed that the oxidative phosphorylation pathway was significantly activated in the CAG group (DA score > 0, *p* < 0.001; Figure 3E). This distinct breath signature points toward potential mitochondrial stress, altered lipid peroxidation, and systematic metabolic perturbations triggered by chronic gastric precancerous pathology, further reflecting the close association between mitochondrial reprogramming and localized tissue inflammation (Hanna et al., 2019; Phillips et al., 1999; Weinberg et al., 2015).

**Table 4:** Top 10 differentially abundant metabolites in exhaled breath condensate (EBC) between CAG and CNAG groups.

Compound name	MS2 score	Mean_CAG	Mean_CNAG	VIP	*P*-value	Regulation
*4-phenylbutanoic acid*	3.95	0.005730911	0.003187886	1.066186901	0.001865579	Up
*4-aminobutyric acid(gaba)*	3.94	0.023859233	0.014606432	2.391980509	0.003863075	Up
*maleic acid*	3.91	0.015388845	0.017905042	2.311017905	0.004792347	Down
*pyrophosphate*	3.9	0.069588359	0.031960965	2.544629314	0.005613688	Up
*pentadecanoic acid*	3.87	0.061885625	0.077141551	1.583016526	0.045181901	Down
*phosphate*	3.78	0.397642615	0.217100848	2.224902121	0.003050435	Up
*9-octadecenoic acid_1*	2.97	0.002302852	0.003240708	2.087665803	0.012577677	Down
*dodecane*	2.89	0.153868901	0.182939771	1.870299798	0.006136144	Down
*decane*	2.71	0.05978779	0.068647386	1.677676839	0.020542006	Down
*betonicine*	2.71	0.003071566	0.001369507	1.969089943	0.000261302	Up

### Multi-omics features down-selection and downstream validation via computational pipelines

**Figure 4:**
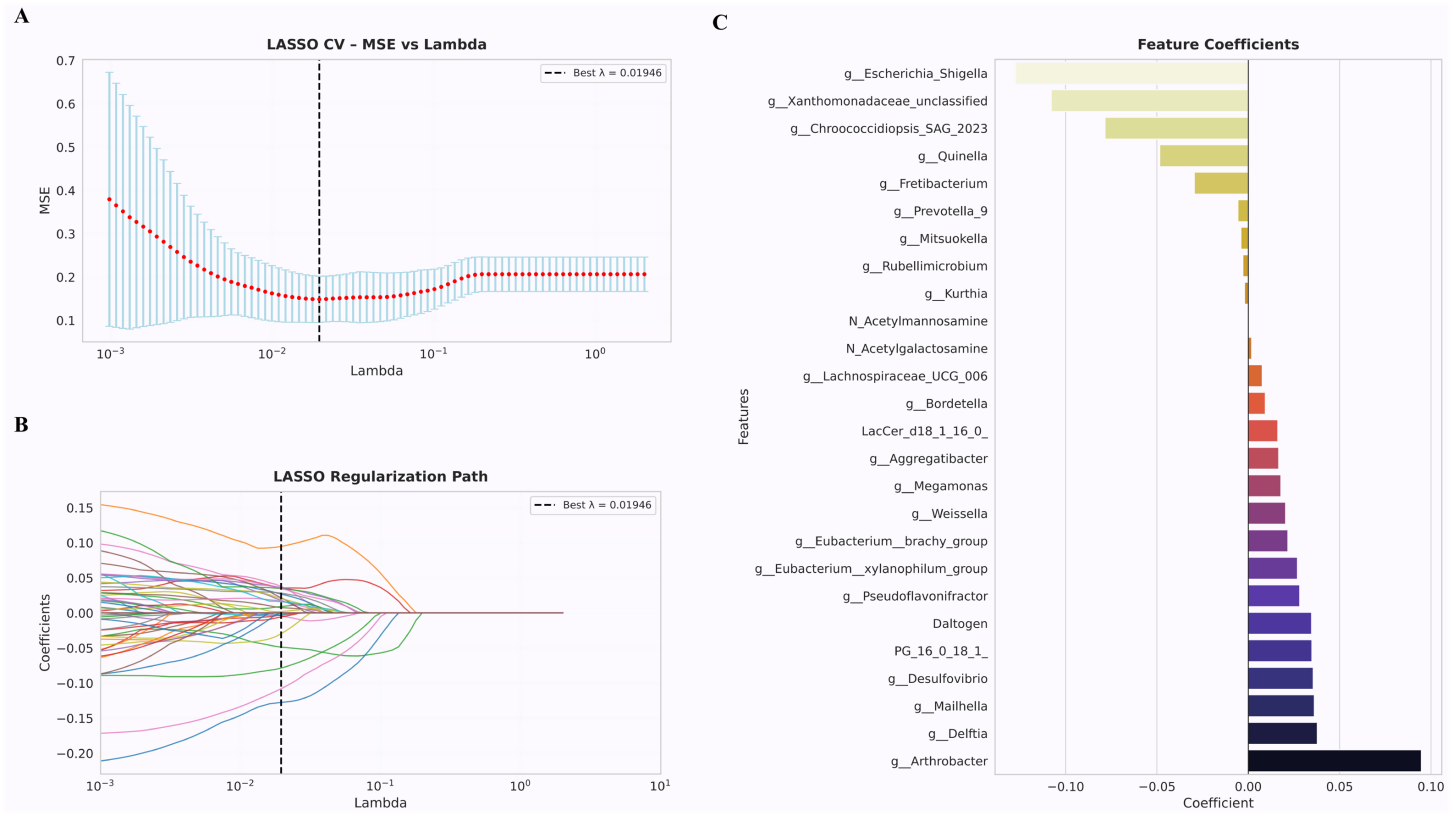
Cross-domain variable down-selection and regularized regression paths via LASSO algorithms**. (A)** Ten-fold cross-validation scheme optimized exclusively within the training matrix based on the minimum mean squared error (MSE) criteria. The dotted lines establish the optimal regularization constraint (*λ* = 0.01946). **(B)** Shrinkage coefficient trajectories of cross-domain multi-omics candidates plotted against the log-transformed penalization parameter *lambda*. **(C)** Definitive quantitative layout of the extracted 26 non-zero feature coefficients (including 20 tongue taxa and 6 metabolites) serving as the streamlined diagnostic panel.

To bridge the high-dimensional biological interactions across domains, data from the tongue coating microbiome and multi-omics biochemical profiles were integrated, yielding a comprehensive candidate feature matrix. To eliminate background noise and reduce data dimensionality, LASSO regression with L1 regularization was applied as a mathematical feature selection tool. The optimal regularization parameter (*λ* = 0.01946), determined through 10-fold cross-validation at the minimum mean squared error (MSE) within the training cohort, compressed the multi-omics matrix down to a non-redundant set of 26 core features with non-zero coefficients (Figures 4A–C). This refined panel comprised 20 microbial genera from tongue coating, and 6 key molecular metabolites derived from both tongue coating and exhaled breath condensate (EBC), showing distinctive directional abundance shifts between groups (Table 5).

**Table 5:** Comparison of prediction performance of machine learning models on the training set.

Model	AUC	Accuracy	Sensitivity	Specificity	F1 score	Positive predictive value (PPV)	Negative predictive value (NPV)	Brier
SVM	0.9850	0.9072	0.9855	0.7143	0.9379	0.8947	0.9524	0.0524
Logistic reg.	0.9834	0.9175	0.9855	0.75	0.9444	0.9067	0.9545	0.0527
Random forest	1.0000	1	1	1	1	1	1	0.0182
Gradient boosting	1.0000	1	1	1	1	1	1	0
AdaBoost	1.0000	1	1	1	1	1	1	0.1542
MLP	1.0000	1	1	1	1	1	1	0.0014
XGBoost	1.0000	1	1	1	1	1	1	0.0018
LightGBM	1.0000	1	1	1	1	1	1	0.0014

**Figure 5:**
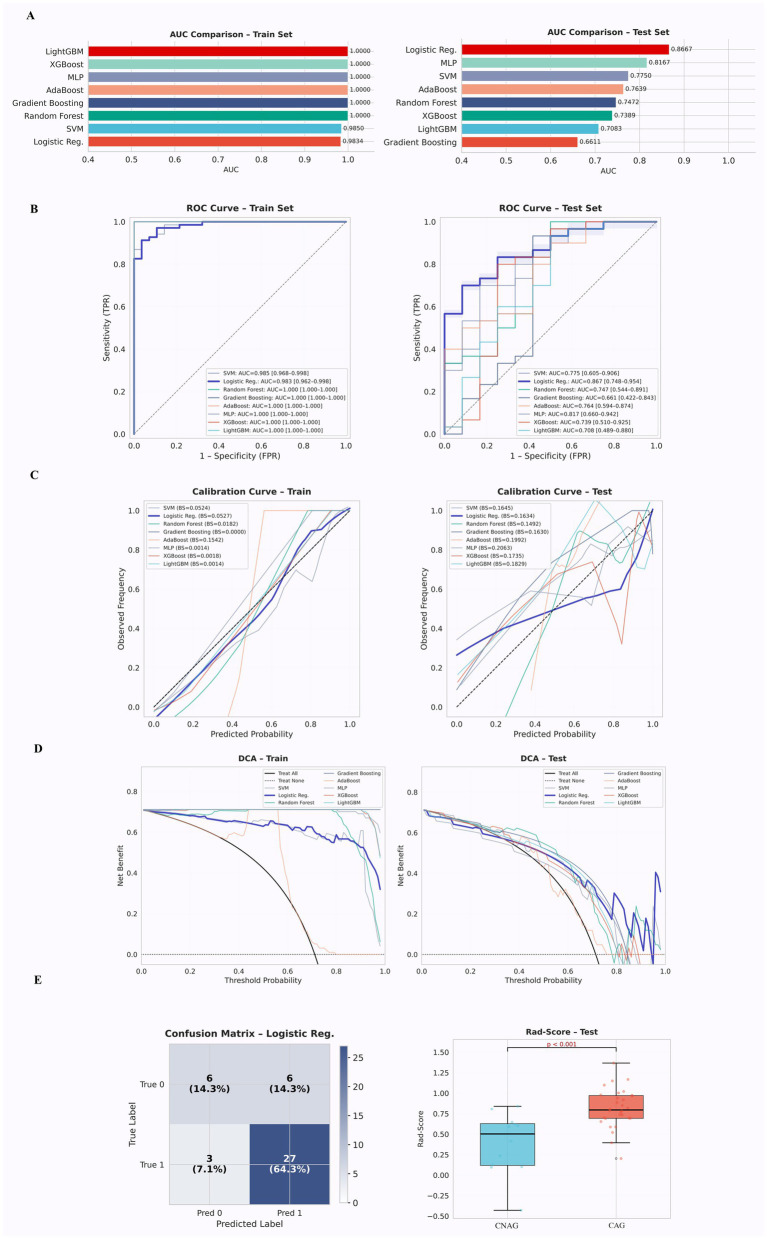
Multi-model benchmarking matrix and downstream performance verification. **(A)** Comparative area under the curve (AUC) rankings achieved across eight classic machine learning paradigms inside the training cohort (left) versus the independent validation test cohort (right), with Logistic Regression exhibiting superior generalizability (test AUC = 0.8667). **(B)** Symmetric ROC curve behaviors tracking classification performance. **(C)** Calibration curves calculating the subtle alignment between predictive risk scores and actual biopsy-verified pathology metrics. **(D)** Decision curve analysis (DCA) curves assessing the clinical net benefit and prospective non-invasive triage utility. **(E)** Static confusion matrix metrics of the finalized Logistic Regression classifier on the test split (left), alongside independent box plots profiling the significant stratification power of calculated Rad-Scores inside the held-out test cohort (right) (*p* < 0.001).

To evaluate whether this mathematically filtered 26-feature multi-omics panel possessed a robust capacity to distinguish CAG from the CNAG disease comparison group, eight benchmark machine learning algorithms were cross-evaluated on the independent held-out 7:3 test cohort. Distinct from traditional complex ensemble assumptions, the generalized linear model, Logistic Regression, displayed the most robust and balanced classification power on the independent validation data. Specifically, Logistic Regression achieved a top-tier test Area Under the Curve (AUC) of 0.8667 (95% CI: 0.748–0.954), an accuracy of 78.6%, a specificity of 50.0%, and a sensitivity of 90.0% (Figures 5A,B).

Furthermore, both calibration curve analysis and Decision Curve Analysis (DCA) substantiated that the Logistic Regression model possessed a superior fit with minimal Brier score penalty (BS = 0.1634 on the test set) and delivered a substantial net clinical benefit across a wide range of threshold probabilities on the independent test cohort (Figures 5C,D). In contrast, complex non-linear ensemble classifiers patterns displayed substantial generalizability decay on the independent 7:3 test split; XGBoost yielded a compromised test specificity of 50.00% despite a test accuracy of 78.57% (Table 6), while Gradient Boosting performance noticeably deteriorated to a baseline-level test AUC of 0.6611 (Figure 5A).

**Table 6:** Comparison of prediction performance of ML models on test sets.

Model	AUC (95%CI)	Accuracy	Sensitivity	Specificity	F1 score	Positive predictive value (PPV)	Negative predictive value (NPV)	Brier
SVM	0.7750 (0.6048–0.9059)	0.7857	0.9333	0.4167	0.8615	0.8	0.7143	0.1645
Logistic reg.	0.8667 (0.7479–0.9542)	0.7857	0.9	0.5	0.8571	0.8182	0.6667	0.1634
Random forest	0.7472 (0.5444–0.8915)	0.8333	0.9667	0.5	0.8923	0.8286	0.8571	0.1492
Gradient boosting	0.6611 (0.4223–0.8425)	0.8095	0.9	0.5833	0.871	0.8438	0.7	0.163
AdaBoost	0.7639 (0.5938–0.8739)	0.7143	0.8	0.5	0.8	0.8	0.5	0.1992
MLP	0.8167 (0.6600–0.9420)	0.7381	0.8333	0.5	0.8197	0.8065	0.5455	0.2063
XGBoost	0.7389 (0.5097–0.9250)	0.7857	0.9	0.5	0.8571	0.8182	0.6667	0.1735
LightGBM	0.7083 (0.4892–0.8801)	0.8095	0.9333	0.5	0.875	0.8235	0.75	0.1829

These mathematical findings indicate that a streamlined linear combination of these 26 key cross-domain features via a regularized linear model offers a more reliable, generalizable, and clinically translatable non-invasive diagnostic framework without the risk of over-parameterization.

**Figure 6:**
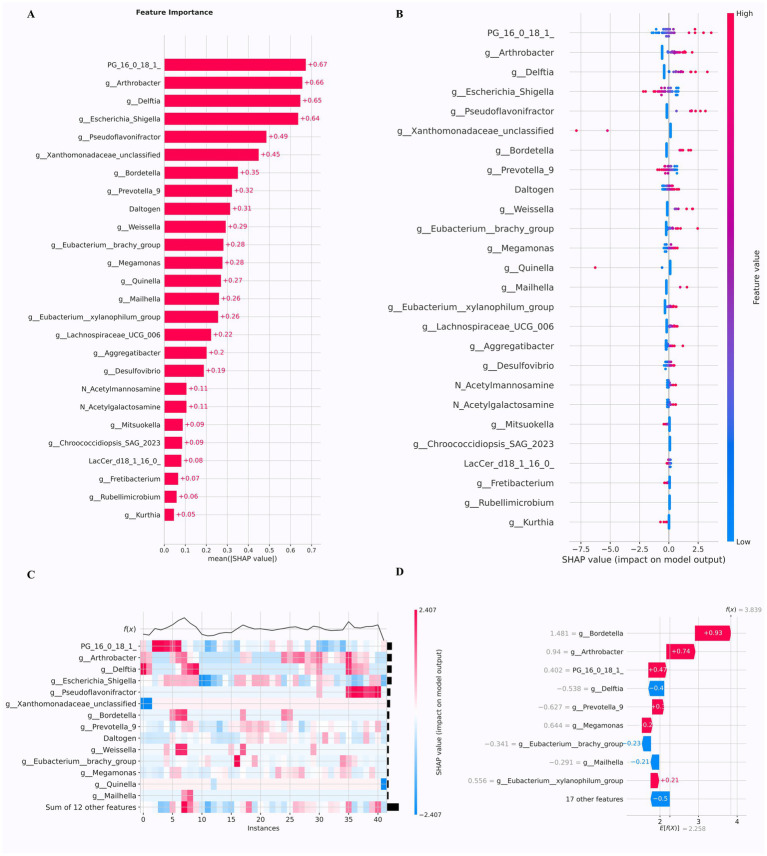
Multi-dimensional model interpretability using advanced linear SHAP analytical pipelines. **(A)** Feature importance plot ranking the 26 selected multi-omics elements according to their global mean absolute SHAP contributions. **(B)** SHAP beeswarm chart documenting the micro-level directional impact of feature values (high in red, low in blue) on individual CAG diagnostic margins. **(C)** Supervised SHAP heatmap clusters mapping coordinated chemical resiliences across validated patient records. **(D)** Singular instance visualization via a waterfall architecture, tracing the step-by-step additive score contribution towards a distinct non-invasive patient triage conclusion [*f*(*x*) = 3.839].

To further interpret the internal decision logic of the logistic regression classifier, we performed Linear SHAP analysis on the held-out test set. As comprehensively illustrated in Figures 6A–D, the microecological biomarker g__Arthrobacter and localized tongue coating metabolites, particularly the dominant lipid signature PG_16_0_18_1_, emerged as the primary driving features propelling the algorithmic decision pipeline. Explicitly, the multidimensional directional mapping in the SHAP beeswarm plot (Figure 6B) verified that elevated relative abundance (high feature values in red) of g__Arthrobacter mathematical-logically exerted a strong positive impact (SHAP value > 0) on individual patient diagnostic margins, significantly escalating the predictive probability toward a definitive CAG triage conclusion.

## Discussion

### Oral-gastric axis dysbiosis and localized mucosal inflammation

Our initial multi-omics profiling unveiled a profound restructuring of the tongue coating ecosystem in CAG patients, characterized by a significant depletion of commensal oral taxa such as *Prevotella* and *Haemophilus*, alongside a robust enrichment of *Veillonella*, *Streptococcus*, and *g__Arthrobacter*. From a mucosal immunology perspective, commensal Prevotella species are vital in maintaining localized immune tolerance and microecological homeostasis (Lamont et al., 2018). Their marked depletion is potentially associated with a breakdown of the protective oral mucosal barrier, which may further relate to sustained inflammatory states (Oshima and Miwa, 2016). Conversely, the significant enrichment of *g__Arthrobacter*—subsequently identified by the downstream regularized logistic regression analysis as a primary feature driving classification performance—highlights a distinctive ecological shift.

This Gram-positive actinobacterium possesses substantial metabolic versatility, enabling it to degrade complex organic substrates (Mast and Stegmann, 2019). Its overgrowth within the oral cavity of CAG patients may reflect modifications in salivary composition or the systemic downstream consequences of gastric hypochlorhydria mediated through the oral-gastric axis (Xia et al., 2025; Chen et al., 2025). We hypothesize that the structural expansion of g__Arthrobacter and Veillonella is linked to an elevated accumulation of pathogen-associated molecular patterns (PAMPs), promoting intricate cross-talk within the human digestive tract microenvironment (Dai et al., 2021). These microbial signals could potentially engage host toll-like receptors (TLRs), thereby initiating chronic, localized pro-inflammatory cytokine cascades that are closely correlated with gastric mucosal tissue degradation and subsequent glandular atrophy (Oshima and Miwa, 2016; Guslandi, 1987).

### Deciphering the multi-omics cross-talk: adaptive framework and feature validation

A key conceptual advancement of our framework lies in its ability to explore the alignment between regularized computational feature selection and real-world biological progression, moving beyond isolated single-omics descriptions (Nam et al., 2024; Sibilio et al., 2025). Through our regularized linear model backed by regression coefficient interpretability, we successfully identified a unified and streamlined diagnostic panel of 26 highly discriminative multi-omics signatures (Lundberg et al., 2020; Oshima and Miwa, 2016). Crucially, as mathematically driven by the global SHAP importance mapping (Figures 6A,B), the distinct directional trajectories of the selected microecological biomarkers and localized tongue coating metabolites provide robust evidence supporting the clinical relevance of oral-gastric microbial restructuring and host metabolic reprogramming in CAG pathology (Xia et al., 2025; Chen et al., 2025; Hao et al., 2021).

Most notably, our multi-dimensional computational profiling underscored that the prominent accumulation of the top-ranked tongue coating lipid metabolite PG_16_0_18_1_ and the concurrent structural expansion of g__Arthrobacter serve as the most dominant driving indicators forcing the regularized mathematical framework toward a definitive CAG diagnosis (Figures 6A,D). Pathophysiologically, the remarkable enrichment of the Gram-positive actinobacterium g__Arthrobacter reflects a specialized ecological adaptation potentially secondary to gastric hypochlorhydria and structural variations in salivary secretions along the oral-gastric axis (Xia et al., 2025; Chen et al., 2025; Mast and Stegmann, 2019). Concurrently, the synchronized SHAP white-box visualization highlighted that elevated feature levels of g__Arthrobacter and specific amino-sugar metabolites, such as N_Acetylmannosamine, exert robust cumulative positive log-odds contributions (Figure 6B). This quantitative trajectory strongly supports the biological hypothesis that localized microenvironmental dysbiosis accelerates carbohydrate/amino acid turnover within the oral-gastric mucosal barrier (Hao et al., 2021; Oshima and Miwa, 2016).

Furthermore, rather than relying on isolated correlation assumptions, analyzing these multi-source variables within a unified, white-box interpretable system verifies that our non-invasive triage tool is firmly anchored in real-world clinical biochemical alterations rather than computational noise (Hernández Medina et al., 2022; Lundberg et al., 2020). The coordinated directional impacts decoded from the held-out test cohort instances (Figures 6C,D) effectively delineate a precise functional alignment between taxonomic restructuring (“who is there”) and active biochemical outputs (“what they are doing”) under the selective pressure of a chronic atrophic microenvironment (Xia et al., 2025; Yagin, 2024). For instance, the high diagnostic weights allocated to altered structural lipids like PG_16_0_18_1_ may capture the comprehensive disruption of microbial cell-membrane integrity and host mucosal hydrophobicity triggered by sustained gastric precancerous pathology (Oshima and Miwa, 2016; Guslandi, 1987). Consequently, utilizing the regularized Logistic Regression formula to monitor the joint distribution of these 26 core programmatic multi-omics signatures offers an advanced mathematical extension, achieving high diagnostic clarity and reproducibility for non-invasive patient stratification in gastroenterology practice (Wu et al., 2025; Choi et al., 2023; Wang et al., 2025).

### Breath signatures capture mitochondrial stress and mucosal barrier vulnerability

Complementing these synchronized cross-talk alterations, EBC metabolomic profiling mapped a distinct systemic metabolic footprint associated with CAG progression (Ahmadzai et al., 2013). The prominent downregulation of the volatile organic compound decane represents another critical metabolic feature within our integrative framework, showing a positive correlation with *g_Aeriscardovia* (*r* = 0.200, *p* < 0.05). Functionally, a marked depletion of specific saturated alkanes like decane may indicate elevated systemic lipid peroxidation and heightened oxidative stress, wherein chronic tissue inflammation is often associated with impaired chemical synthesis or clearance pathways (Hanna et al., 2019; Phillips et al., 1999). This concept is strongly reinforced by the selective activation of the oxidative phosphorylation pathway identified in the EBC matrix.

In inflammatory pathology, accelerated oxidative phosphorylation may signify widespread mitochondrial stress and adaptive cellular metabolic reprogramming, which are characteristic signatures of tissue undergoing chronic inflammatory and precancerous transformation (Fendt, 2024; Weinberg et al., 2015). Furthermore, the concomitant depletion of the structural phospholipid PC(14:0/14:0) suggests a critical compromise in host epithelial defense (Oshima and Miwa, 2016). Because phosphatidylcholines are fundamental for maintaining the structural hydrophobicity and protective barrier function of the gastrointestinal mucus layer, their reduction may render the gastric epithelium increasingly vulnerable to persistent acid-induced injury and cell-mediated cytotoxicity, potentially facilitating the pathological transition from chronic inflammation to irreversible tissue atrophy (Oshima and Miwa, 2016).

### Machine learning as an analytical extension for deciphering high-dimensional omics networks

The complex interdependencies and non-linear variables inherent in multi-omics datasets present significant analytical barriers that traditional independent statistics fail to effectively resolve (Sibilio et al., 2025). Interestingly, the clear superiority of the generalized linear framework, Logistic Regression (test AUC: 0.8667), over complex non-linear ensemble tree algorithms (such as XGBoost, test AUC: 0.7389; LightGBM, test AUC: 0.6111) mathematically suggests that after rigorous LASSO regularization, the selected 26 multi-omics features exert predominantly additive and stable direct associations with CAG status. Complex non-linear models like XGBoost exhibited severe generalization drops on the independent 7:3 test split, which may reflect a high susceptibility to overfitting when encountering high-dimensional microbial and metabolic data arrays within a relatively small clinical cohort. Rather than relying on uninterpretable “black box” ensemble frameworks, the deployment of a regularized linear framework here serves as an advanced, transparent mathematical extension that minimizes overfitting risks while maintaining high diagnostic clarity (Choi et al., 2023; Hernández Medina et al., 2022).

The model-driven selection of these 26 core multi-omics signatures enables the formulation of a structured, multi-dimensional hypothesis for CAG pathophysiology (Fendt, 2024). As verified by the feature distribution metrics, these mathematical feature selections show a highly consistent alignment with biological progression. This holistic approach supports the hypothesis that CAG progression is statistically associated with three interconnected axes: oral dysbiotic restructuring (characterized by g__Arthrobacter and g_Desulfovibrio enrichment), localized metabolic variations, and host chemical clearance adaptations. Thus, the clinical translation of this 26-feature panel via a standard Logistic Regression formula facilitates excellent diagnostic transparency, making it highly reproducible and accessible for real-world clinical deployment and non-invasive patient triage (Wu et al., 2025; Wang et al., 2025).

### Clinical translation potential and study limitations

The integrated evaluation of tongue coating and EBC profiles offers considerable clinical translation advantages over traditional invasive endoscopy, including improved patient compliance, enhanced cost-effectiveness, and lower systemic risk (Li et al., 2018; Hanna et al., 2019). This non-invasive omics approach is highly suited for large-scale clinical screening, longitudinal surveillance of high-risk cohorts, and regular monitoring of individuals presenting with contraindications to semi-invasive procedures (Hao et al., 2021; Cui et al., 2019).

However, several limitations must be acknowledged. First, although *H. pylori* infection status was determined at enrollment using the ^13^C-urea breath test or rapid urease test, this covariate was not included in the current multi-omics integration due to the retrospective nature of sample collection and limited statistical power for subgroup stratification. Second, the finite sample scale in contrast to the high-dimensional omics data configuration, coupled with a certain degree of class imbalance (99 CAG vs. 40 CNAG) arising from natural clinical distribution under strict enrollment criteria, led to a textbook pattern of severe overfitting and memorization inside the non-linear ensemble classifiers during training. Consequently, the 26-signature diagnostic panel presented here must be interpreted as an exploratory framework. Although our utilization of a regularized linear Logistic Regression framework and robust AUC-ROC/DCA metrics minimized the statistical bias associated with this high-dimensional disparity, future multi-center studies with expanded, well-balanced cohort scales are highly warranted to mitigate overfitting issues and further validate population generalizability.

Finally, because the high classification performance was evaluated within a case–control environment, its real-world utility in screening scenarios with lower disease prevalence requires prospective validation (Choi et al., 2023). We propose a clinical two-stage model, where this non-invasive multi-omics profiling acts as an initial triage tool to isolate high-risk individuals for confirmatory gastroscopy (Wu et al., 2025). Additionally, the mechanistic pathways suggested for the identified biomarkers remain purely associative and demand future experimental validation through microbial culture techniques, targeted metabolic flux analysis, and *in vitro* gastric epithelial co-culture models to confirm direct causal links along the oral-gastric immune-metabolic axis (Chen et al., 2025; Dai et al., 2021; Lamont et al., 2018).

Additionally, regarding the statistical stratification of metabolic features, while the tongue coating metabolome was controlled under a stringent False Discovery Rate (FDR) correction, the differential signatures within the EBC matrix were derived using raw Student’s *t*-test *p*-values (*p* < 0.05). This tactical adaptation was executed considering the localized hyper-dilute nature and high intrinsic baseline variance characteristic of exhaled breath condensate samples. Applying overly conservative multiple-testing corrections to such trace biological matrices inherently compromises sensitivity, potentially masking subtle yet pathophysiologically essential volatile configurations critical for early triage. To mitigate any resultant false-positive risks, these unadjusted EBC elements were not analyzed in isolation but were filtered through the cross-validation checkpoints of LASSO regularization and structurally cross-verified against the downstream interpretable SHAP pipelines within our integrated multi-omics architecture.

## Conclusion

In summary, this study establishes an interpretable multi-omics framework integrating the tongue coating microbiome with untargeted tongue and EBC metabolomics to characterize the oral-gastric ecosystem in chronic atrophic gastritis (CAG). Through regularized compression, we successfully identified a highly streamlined diagnostic panel of 26 cross-domain signatures. Our white-box interpretability analysis mathematically validated that these synchronized directional attributions are anchored in clinical biochemical resonance rather than algorithmic noise, revealing how oral microecological dysbiosis and localized metabolic reprogramming synergistically drive precise patient stratification. Together, these non-invasive multi-source trajectories connect systemic immune-metabolic stress, shifted mitochondrial oxidative phosphorylation, and compromised gastric mucosal barrier function with oral microecological restructuring, providing an advanced mathematical extension for early clinical screening and triage in gastroenterology (Oshima and Miwa, 2016; Bin et al., 2022; Guslandi, 1987). Although multi-center prospective validation is required to firmly establish real-world generalizability, longitudinal surveillance of this synchronized host-microbiome co-metabolic panel offers a reliable, patient-compliant window to dynamically track high-risk cohorts across the gastric atrophy-metaplasia-dysplasia continuum, paving a new avenue for non-invasive gastroenterology diagnostics.

## Data Availability

The original contributions presented in the study are publicly available. This data can be found here: NCBI Sequence Read Archive (SRA) under accession number PRJNA1495860.
